# BAP1 Loss in Pleural Mesothelioma Is Associated With Reduced Soluble CCL2 in Patient Effusion, Abrogated CCL2-Mediated Monocyte Recruitment In Vitro

**DOI:** 10.1016/j.jtocrr.2026.101049

**Published:** 2026-07-10

**Authors:** Alistair Nash, Ebony Rouse, Tina Firth, Ian M. Dick, Vanessa S. Fear, Catherine A. Forbes, Amber Louw, Y.C. Gary Lee, Alec J. Redwood, Bruce W. Robinson, Jenette Creaney

**Affiliations:** a National Centre for Asbestos Related Diseases, University of Western Australia, Perth, Australia; b Medical School, University of Western Australia, Perth, Australia; c Institute for Respiratory Health, Perth, Australia; d The Kids Research Institute, University of Western Australia, Perth, Australia; e Department of Anatomical Pathology, PathWest Laboratory Medicine, QEII Medical Centre, Perth, Australia; f Department for Respiratory Medicine, Sir Charles Gairdner Hospital, Perth, Australia

**Keywords:** Pleural, Mesothelioma, BAP1, Effusion, CCL2, Monocytes

## Abstract

**Objectives:**

Pleural mesothelioma is an incurable cancer of the cell layer lining the chest wall and lung. Patients frequently present with pleural effusion, which is often drained for symptom relief and enables minimally invasive sampling of the tumour environment, including immune cells and related soluble factors. Most of the mesothelioma tumours exhibit loss of BRCA1-associated protein 1 (BAP1), a multifunctional tumour suppressor protein. Here, we aim to elucidate the effect of BAP1 loss on the mesothelioma microenvironment through profiling soluble factors within pleural effusion.

**Methods:**

A custom panel of 22 soluble factors was measured by Luminex assay and enzyme-linked immunosorbent assay in an initial cohort of 40 patients with known BAP1 status. Validation was performed by enzyme-linked immunosorbent assay in an independent cohort of 100 cases. Secretion of soluble factors and chemoattraction of monocytes were characterised using a CRISPR-mediated BAP1 deletion model in a mesothelioma and a lung cancer cell line. Immune cell infiltration, estimated by CIBERSORT, was further explored in the Cancer Genome Atlas -MESO cohort.

**Results:**

Soluble C–C motif chemokine ligand 2 (CCL2) was approximately 55% to 60% lower in pleural effusion from BAP1-loss cases in both independent cohorts. Deletion of BAP1 reduced CCL2 secretion in vitro and abolished CCL2-mediated chemoattraction of monocytes in both mesothelioma and lung cancer cell lines. In the Cancer Genome Atlas -MESO cohort, BAP1-mutant tumours exhibited a reduction in estimated macrophage content.

**Conclusion:**

Loss of BAP1 impairs CCL2 secretion into pleural effusions, potentially influencing monocyte recruitment into the tumour microenvironment.

## Introduction

Pleural mesothelioma is an aggressive, asbestos-related cancer of the mesothelial cells surrounding the lung. Mesothelioma has a poor prognosis, with a median survival after diagnosis of approximately 18 months, and current therapies remain noncurative.^1^ The majority of individuals with mesothelioma present with pleural effusion, an accumulation of fluid within the pleural cavity^2^ which bathes the tumour in a range of growth factors and inflammatory cytokines. Pleural effusions are heterogeneous in both cellular composition and concentrations of soluble factors.^3^^,^^4^

Previously, we have reported that effusions contribute to mesothelioma pathobiology both in vitro and in vivo; mesothelioma pleural fluid was found to promote cell proliferation and migration and to increase resistance to chemotherapeutic agents.^3^ More recently, we reported that the profile and concentration of cytokines and chemokines in mesothelioma effusions are predictive of patient prognosis.^4^

*BAP1* (BRCA1-associated protein 1), which encodes a deubiquitinating enzyme that acts as a multifunctional tumour suppressor, is one of the most frequently mutated genes in mesothelioma. BAP1 loss has been associated with alterations in the tumour immune environment across multiple cancer types. In uveal melanoma and renal carcinoma, BAP1-deficient tumours exhibit increased intratumoural lymphocyte and macrophage infiltration.^5^, ^6^, ^7^ In mesothelioma, CD8^+^ T cell infiltration was increased in BAP1-mutant epithelioid tumours.^8^ Mechanistically, BAP1 loss enhances the secretion of the proinflammatory mediator high-mobility group box 1 (HMGB1) from damaged cells.^9^ These findings point to a role for BAP1 in modulating inflammatory signalling through HMGB1 release. However, broader changes to the cytokine milieu associated with BAP1 loss in mesothelioma remain largely inferred from transcriptomic data, and the direct measurement of soluble immune mediators in clinical samples is limited. To address this, we investigated whether BAP1 status correlates with cytokine and chemokine levels in pleural effusions, fluids that contain a heterogeneous mixture of soluble factors that reflect the tumour microenvironment.

In this study, we focus on a panel of 22 soluble markers previously identified as either associated with BAP1 status or as independent predictors of survival in our prior work. These include HMGB1 and the C-C motif ligand 2 (CCL2), a key chemokine involved in recruiting cells of the monocyte-macrophage lineage. By elucidating the association between BAP1 status and effusion cytokine profiles, we aim to advance understanding of the immunobiology of mesothelioma and identify potential avenues for therapeutic intervention.

## Methods

### Study Design and Participants

Consecutive patients attending a specialist referral centre for mesothelioma were invited to participate in the National Centre for Asbestos Related Diseases Biobanking program. All subjects provided written informed consent to participate in research, and ethical approval was granted by the Sir Charles Gairdner and Osborne Park Health Care Group and the University of Western Australia Human Research Ethics Committees. This research was conducted in accordance with the Declaration of Helsinki. Eligible subjects had a pathologically confirmed diagnosis of pleural mesothelioma, with demographic and clinical data available. Some patients included in the present study had been previously reported.^4^ Tumour BAP1 protein expression was determined by immunohistochemistry using anti-BAP1 C-4 mouse monoclonal antibody (Santa Cruz Biotechnology), as previously described.^10^ The censoring date for patient survival analysis was May 27, 2025.

### Sample Selection

Subjects were eligible for inclusion if they had a pleural effusion sample collected within 10 days of the pathology specimen assessed for BAP1 status. Samples were excluded if the patients had received systemic anti-cancer therapy before effusion collection. Sample selection was independent of patient survival.

For the discovery cohort, 40 pleural effusion samples were selected based on *BAP1* status to achieve equal numbers of cases with *BAP1* loss (n = 20) and BAP1 retention (n = 20). Within each group, samples were randomly selected. This approach enriched for BAP1 retention, which is less common in unselected mesothelioma cohorts. A further 100 pleural effusions (55 with BAP1 loss and 45 with *BAP1* retention) formed the validation cohort, selected using the same criteria. Effusion samples were processed as previously described.^4^

For paired serum analyses, 22 serum samples were selected from patients who had matched pleural effusions collected within 40 days, provided that no systemic anti-cancer treatment or pleurodesis occurred within that interval.

### Custom 21-Plex Luminex Assay

Concentrations of 21 soluble factors were determined in pleural effusion supernatants using a custom-designed human magnetic Luminex 21-plex assay (R&D Systems). Analytes were selected from putative immune-related factors proposed to be associated with BAP1 expression in the research literature or previously associated with prognosis^4^ (Supplementary Table 1). The assay was performed according to the manufacturer’s instructions, with all samples diluted 1-in-2 and analysed in duplicate. Raw fluorescence data were fitted to a 5-parameter logistic curve using Belysa version1.1.0 software (Sigma-Aldrich, St. Louis, MO), and concentrations were determined. Results below or above the range of the standard curve were extrapolated.

### Enzyme-Linked Immunosorbent Assay

CCL2 concentrations in pleural effusion, serum, and supernatant were measured using the R&D Systems DuoSet enzyme-linked immunosorbent assay (ELISA) kit (R&D Systems). Samples were diluted 1:5 for pleural effusion and serum and 1:2 for supernatant. HMGB1 concentrations in pleural effusions from the discovery cohort were quantified separately using a human HMGB1 ELISA kit (Antibodies-Online) with a 1:20 dilution due to Luminex incompatibility. ELISA assays were performed according to the manufacturer’s instructions, with samples analysed in duplicate. Absorbances were fit to a 4-parameter standard curve to calculate analyte concentrations.

### Cell Culture

The human mesothelioma cell line JU77^11^ and the lung cancer cell line A549 (ATCC) were cultured in RPMI 1640 (Thermo Fisher Scientific) containing 10% foetal calf serum (FCS, Invitrogen) and 20 mM HEPES (Sigma-Aldrich). JU77 and A549 both retain wild-type BAP1 and are deficient in Cyclin-Dependent Kinase Inhibitor 2A. Neurofibromatosis 2 is lost in JU77 but retained in A549. Cells were passaged and harvested using 0.25% trypsin (Sigma-Aldrich).

For the collection of cell culture supernatants, cells were seeded at 10^5^ cells per well in 12-well plates with 1 mL of media. Supernatants were harvested at the specified time points, centrifuged at 300*g* for 3 minutes, filtered through a 0.4 μm pore strainer, and stored at -20^o^C until analysis.

### CRISPR-Mediated BAP1 Deletion

BAP1-knockout (BAP1KO) cells were generated from JU77 and A549 parental lines using Alt-R CRISPR/Cas9 tracrRNA ATTO550 (IDT) and custom-designed guide RNAs with XT modification (IDT, Alt-R CRISPR HDR Design Tool). The guide RNA sequences used to target BAP1 were crRNA XT BAP1_AK (-) 5’-CACGGACGUAUCAUCCACCA-GUUUUAGAGCUAUGCU-3’ and crRNA XT BAP1_AN (-) 5’-GCTCTTCGATCCATTTGAAC-GUUUUAGAGCUAUGCU-3’, both with a protospacer adjacent motif restriction site ‘AGG’.

On the day of transfection, cells were seeded at 10^5^ cells per well in a 24-well plate. CRISPR/Cas9 complexes were formed by incubating tracrRNA ATTO 550 with guide RNA, and then further incubating the RNA complex with Cas9. CRISPR/Cas9 complexes were delivered to cells using Lipofectamine CRISPRMAX CAS9 Transfection Reagent (Invitrogen) in Opti-MEM (Gibco).

Single-cell clones were generated by limiting dilution. For JU77 cells, irradiated feeder cells (80 Gy, Gammacell Irradiator 3000, Best Theratronics, Ottawa, Ontario, Canada) were added to support clonal growth. Expanded clones were screened for BAP1 deletion by Sanger sequencing, with genomic sequences analysed using CRISPResso2 (Edilytics, Cambridge, MA). Complete loss of BAP1 protein expression was confirmed by western blotting. Control clones that underwent the CRISPR process but retained wild-type BAP1 sequence were used for BAP1 wild-type (BAP1WT) experiments.

### Western Blot

Cells were lysed using SDS-Page sample protein buffer, boiled at 95^o^C for 10 minutes with reducing buffer, then loaded onto Mini-PROTEAN TGX 4% to 20% precast polyacrylamide gels (BioRad). Proteins were transferred to membranes using a Trans-Blot Turbo Mini 0.2μm Nitrocellulose Transfer Pack (BioRad) and incubated with a 1:1000 HRP-conjugated anti-BAP1 antibody (sc-28383 HRP, Santa Cruz Biotechnology) for 2 hours. As a loading control, an anti-GAPDH primary antibody (#2118, Cell Signalling Technology) followed by an HRP-conjugated anti-rabbit secondary antibody (#7074, Cell Signalling Technology) was used, with incubation for 2 hours for each antibody. Detection was performed using Clarity Max ECL substrate (BioRad), and images were obtained on a ChemiDoc scanner (BioRad).

### Immunofluorescence

Cells grown on coverslips were fixed with 4% formaldehyde (Sigma-Aldrich) for 15 minutes. After each step, the cells were washed with PBS. Permeabilisation was performed with 0.2% Triton-X (Sigma-Aldrich) for 5 minutes, followed by blocking with Background Sniper (Biocare Medical) for 15 minutes. Cells were stained with 1:50 BAP1-FITC (sc-28383 FITC, Santa Cruz Biotechnology) in PBS containing 1% bovine serum albumin (Sigma-Aldrich) for 1 hour at 37^o^C. Coverslips were rinsed with deionised water and mounted on glass slides using Mounting Medium with DAPI (Abcam). Imaging was performed using an Olympus IX81 inverted fluorescence microscope (Olympus Corporation, Tokyo, Japan).

### Monocyte Isolation

Classical monocytes (CD14^+^CD16^-^) were isolated from healthy donor peripheral blood mononuclear cells using a magnetic separation kit (Miltenyi Biotec, Human Classical Monocyte Isolation Kit). The purity of the isolated monocytes was confirmed by flow cytometry after staining with fixable viability dye, CD14-BV510, and CD16-BV786 antibodies (#566332, #563079, and #563789, BD Biosciences). Cells were seeded into a 96-well U-bottom plate at 10^5^ cells per well. Samples were washed twice with FACS buffer (PBS containing 2% FCS), stained for 1 hour with specified antibodies on ice and in the dark, then washed and fixed using the Cytofix/Cytoperm Fixation/Permeabilization kit (BD Biosciences). Samples were acquired on an LSR Fortessa flow cytometer (BD Biosciences, San Jose, CA) and analysed using FlowJo v10.6.2 (BD Biosciences).

### Monocyte Transwell Migration Assay

Freshly isolated classical monocytes were seeded at a density of 10^5^ cells into 8 μm pore transwell inserts (Corning) placed in 24-well plates. Cancer cell supernatant (500 μL) or control medium (RPMI with 0% [R0] or 10% FCS [R10]) was added to the lower chamber. After 6 hours, cells that had migrated to the lower chamber were counted. For CCL2 neutralisation experiments, supernatant was pre-incubated with 4.5 mg/mL recombinant human CCL2 polyclonal antibody (RDSAF279NA, R&D Systems) for 3 hours before assay setup. Cell migration was expressed relative to the R10 control.

### *Estimation of Immune Infiltration in* The Cancer Genome Atlas *-MESO Cohort*

Clinical and genomic data for the Cancer Genome Atlas (TCGA) MESO cohort were obtained through cBioPortal. Only data from the 74 samples used in the final published TCGA-MESO data set were used for analysis.^12^ Patients were sorted by BAP1 mutational status as determined by TCGA. Immune cell composition was estimated using the CIBERSORT algorithm,^13^ implemented through the TIMER2.0 web portal.^14^ Relative abundance scores for immune cell subsets were compared between *BAP1-*mutant and *BAP1*-wildtype tumours.

### Statistical Analysis

Statistics and data analysis were performed using GraphPad Prism (version 9.5.1, GraphPad Software, Boston, MA) and RStudio (2023.09.0, Posit Software, Boston, MA). Descriptive statistics are presented as medians with interquartile range or means with standard deviation, as appropriate. Group comparisons were performed using Student’s *t*-test for normally distributed data and the Mann–Whitney *U* test for nonparametric data. Fisher’s exact test was used to assess differences in categorical variables.

Univariate receiver operating characteristic (ROC) curve analysis for each of the 22 measured variables was calculated using the pROC package in R (v4.3.0). Variables were ranked by AUC, and the top five were included in a support vector machine classification model to assess multivariable predictive performance. Correlation between continuous variables was assessed using Pearson’s correlation coefficient. Heatmaps were generated using the “pheatmap” R package and associated dependencies. Soluble factor concentrations were Z-score normalised, and hierarchical clustering of soluble factors was performed using complete linkage and Euclidean distance. Univariate survival analyses were performed using log-rank testing. Multivariate analyses were performed using Cox regression incorporating BAP1 status, histology, age at diagnosis, sex, Eastern Cooperative Oncology Group (ECOG) performance status at diagnosis, treatment, and soluble CCL2 as covariates. Resulting forest plots were generated using the “survivalAnalysis” R package and associated dependencies.

## Results

### Patient Cohort Characteristics

The discovery cohort comprised 40 patients with pleural mesothelioma, evenly divided between those with BAP1 retention (n = 20) and BAP1 loss (n = 20). The majority were male over 70 years of age, had epithelioid histology, had a good performance status at diagnosis (ECOG 0–1), and subsequently received at least one line of systemic therapy (Table). Fifteen patients received best supportive care, whereas the rest of the cohort was treated with chemotherapy, immunotherapy, or experimental compounds as part of a clinical trial. There were no significant differences between the BAP1-retained and BAP1-loss groups in age, sex, histology, performance status, or treatment received.

**Table:** Discovery and Validation Cohort Patient Characteristics, Grouped by Patient *BAP1* Status

Characteristic	Discovery cohort (n = 40)			Validation cohort (n = 100)		
BAP1 status	BAP1 retained (n = 20)	BAP1 loss (n = 20)	*p* value	BAP1 retained (n = 45)	BAP1 loss (n = 55)	*p* value
Median age (years, IQR)	74 (65-80)	73 (62-76)	0.301^a^	76 (70-81)	73 (61-77)	0.091^a^
Sex			1.000^b^			1.000^b^
Male	19	18		36	45	
Female	1	2		9	10	
Histology			1.000^b^			0.0002^b^
Epithelioid	18	19		22	47	
Non-epithelioid^d^	2	1		23	8	
ECOG			1.000^b^			0.130^b^
0-1	17	18		37	51	
2-3	3	2		8	4	
Therapy			0.515^b^			0.037^b^
Treated	11	14		23	40	
BSC	9	6		22	15	
Median survival (months, 95% CI)	11.9 (7.5-19.4)	11.0 (5.62-23.0)	0.393^c^	10.8 (7.2-11.2)	22.2 (15.6-27.1)	<0.0001^c^

BAP1, BRCA1 Associated Protein 1; IQR, Interquartile range; ECOG, Eastern Cooperative Oncology Group; BSC, Best Supportive Care; CI, Confidence Interval.

a Compared by Mann-Whitney U test.

b Compared by Fisher’s exact test.

c Compared by Log-Rank test.

d Non-epithelioid histology includes biphasic and sarcomatoid subtypes.

### Quantification of Soluble Factors in Pleural Effusion

**Figure 1:**
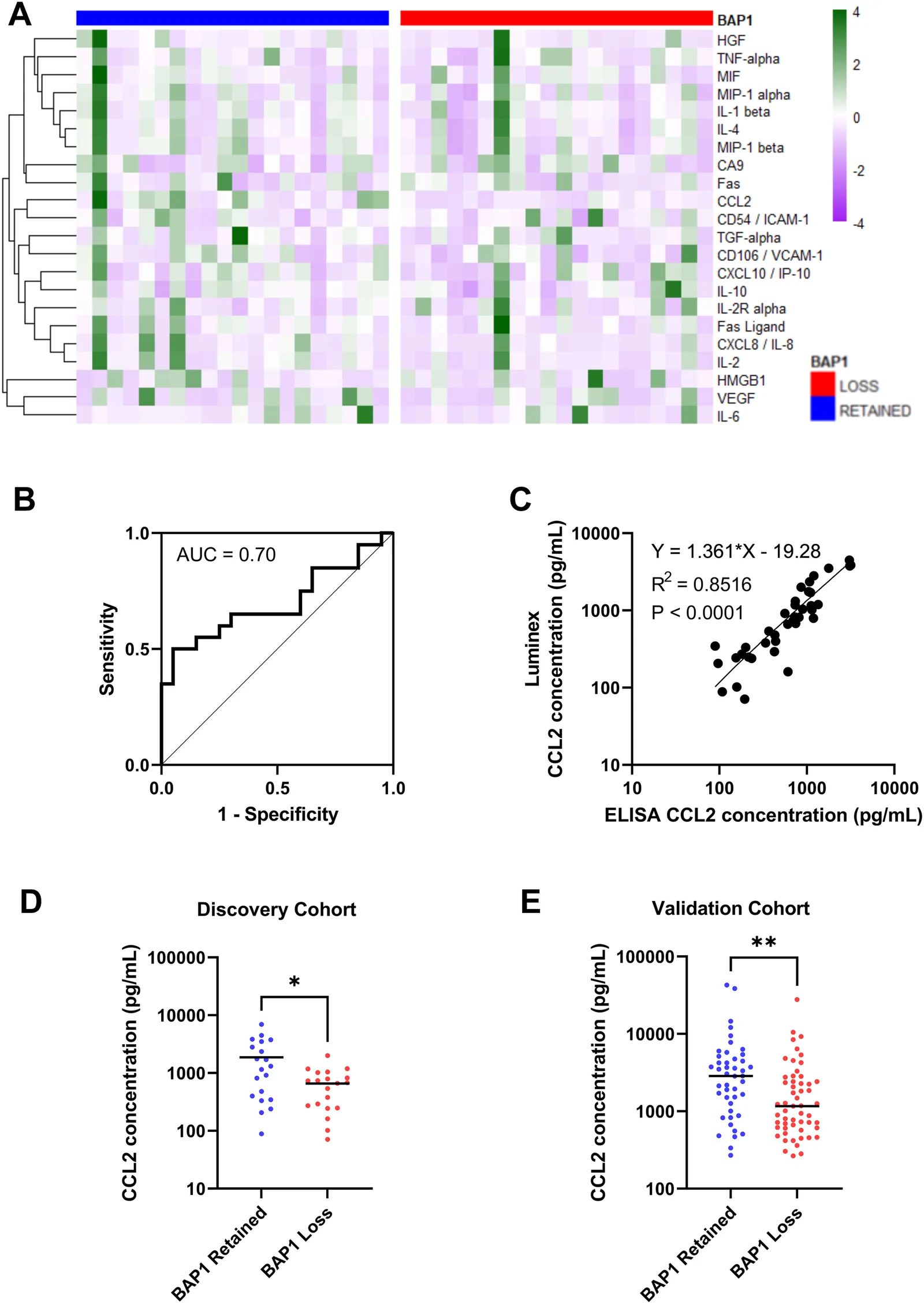
Profiling pleural effusion immune milieu (*A*) Heatmap of concentrations for 22 soluble factors in patient pleural effusions in the discovery cohort (N = 40, BAP1 retained = 20, BAP1 loss = 20) measured by Luminex bead array and ELISA. Soluble factors normalised by Z-score, with supervised clustering by BAP1 status. (*B*) Receiver operating characteristic curve for soluble CCL2 as a predictor of BAP1 status in the discovery cohort. (*C)* Comparison of CCL2 concentrations as measured by Luminex assay and commercial ELISA in discovery cohort samples. (*D*) Column scatter plot of pleural effusion CCL2, measured by commercial ELISA in the discovery cohort (N = 40, 20 BAP1 retained, 20 BAP1 loss), grouped by BAP1 status. (*E*) Column scatter plot of pleural effusion CCL2, measured by commercial ELISA in the validation cohort (N = 100, 45 BAP1 retained, 55 BAP1 loss), grouped by BAP1 status. Compared using the Mann-Whitney test. ∗ *p* < 0.05, ∗∗ *p* < 0.01. BAP1, BRCA1-associated protein 1; CCL2, C-C motif chemokine ligand 2; ELISA, enzyme-linked immunosorbent assay.

Concentrations of 21 soluble factors were determined in 40 pleural effusion samples using a custom Luminex assay (Fig. 1*A*). The data generated were robust, with standard curves for each analyte having an R^2^ greater than 0.99, and for 39 of 40 samples, duplicate assays had a more than 20% coefficient of variation for all analytes. Across the assay, 53 readings (6.3%) were above, and 38 readings (4.5%) were below the limits of the standard curve, and thus these concentrations were extrapolated. HMGB1 concentration could not be determined using the Luminex assay, and therefore these factors were measured in the same samples by ELISA.

### BAP1 Loss is Marked by Lower CCL2 Concentrations in Pleural Fluid

To assess whether soluble mediators in pleural effusion could distinguish BAP1 “Loss” from “Retained” status, we performed univariate ROC analysis across 22 markers. CCL2 was the most predictive individual factor (AUC = 0.70) (Fig. 1*B*), with lower concentrations observed in cases with BAP1 loss. Other top-performing markers included HGF (AUC = 0.67), IL-8 (AUC = 0.67), and IL-2 (AUC = 0.67). A ranked summary of AUCs is shown in Supplementary Table 2.

To assess whether multivariable modelling could improve classification performance beyond that of individual markers, we trained a support vector machine model using the five highest-ranked variables (CCL2, HGF, IL-8, IL-2, and IL-4). However, multivariable modelling did not outperform CCL2 alone (cross-validated AUC = 0.6425), indicating the limited added value of the other analytes. Variable importance confirmed CCL2 as the dominant contributor (importance = 100), with limited additional value from the other features. These findings suggest that CCL2 alone captures most of the signal distinguishing BAP1 status in this data set.

Given the practicality and clinical scalability of ELISA, we assessed its suitability as an alternative method to confirm Luminex results. CCL2 concentrations measured by ELISA in pleural effusion samples showed a strong correlation with Luminex results (R^2^ = 0.85, *p* < 0.0001) (Fig. 1*C*). As measured by ELISA, the concentration of CCL2 was significantly higher in BAP1 retained than BAP1 loss effusions (median [interquartile range]: 1232 [255–969] pg/mL versus 670 [359–3337] pg/mL, *p* = 0.030) (Fig. 1*D*). In matched serum samples from 22 patients, no correlation with pleural effusion levels was observed, and serum CCL2 concentrations did not differ significantly by *BAP1* status (Supplementary Fig. 1*A* and *B*).

To validate the association between BAP1 status and CCL2 levels, we analysed pleural effusion samples from an independent cohort of 100 patients selected using the same inclusion criteria as the discovery cohort. In this validation set, patients with BAP1 retention (n = 45) had a significantly higher proportion of non-epithelioid histology compared with those with BAP1 loss (n = 55) (*p* = 0.0002). No patient underwent surgical resection. Among those receiving systemic therapy, approximately 45% received chemotherapy alone, 20% received immunotherapy alone, and 35% received both modalities. Treatments were administered at the clinician’s discretion, including clinical trial therapies, and were heterogeneous within these categories. A lower proportion of patients with BAP1 retention received systemic therapy (*p* = 0.037). There were no significant differences between the groups in terms of age, sex, and ECOG performance status (Table).

Consistent with findings from the discovery cohort, pleural effusion CCL2 concentrations measured by ELISA were significantly higher in the BAP1-retained group compared with the *BAP1*-loss group (2864 [1133–4944] versus 1163 [612–2421] pg/mL, *p* = 0.0025; Fig. 1*E*).

**Figure 2:**
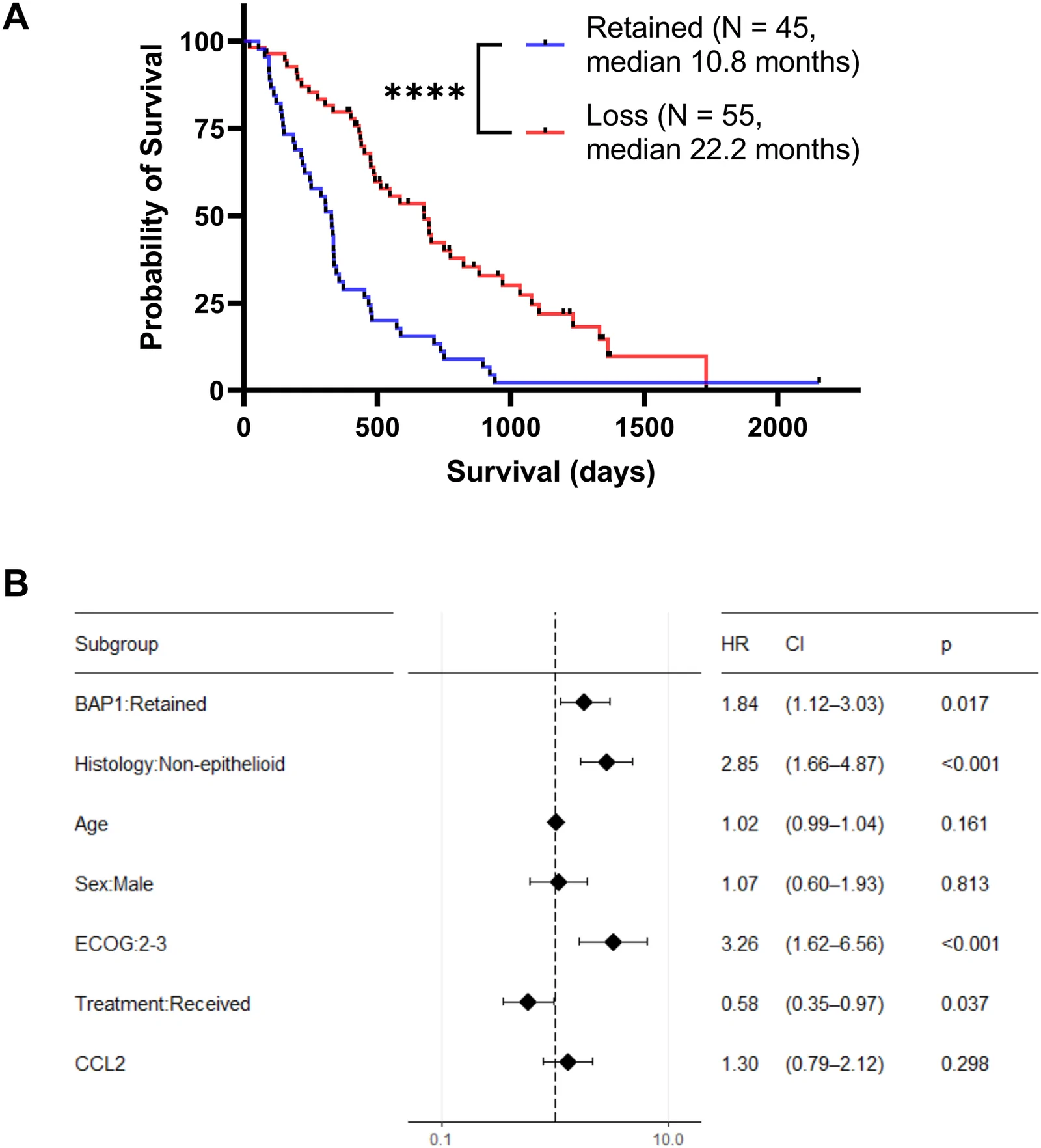
Patient survival in the validation cohort. (*A*) Survival of validation cohort patients with BAP1 retention (N = 45) or BAP1 loss (N = 55), assessed by the Log-Rank test. (*B*) Forest plot showing multivariate Cox regression analysis, assessing the association of clinical characteristics, BAP1 status and pleural effusion CCL2 concentration with survival. HR – Hazard Ratio, CI – 95% confidence interval, ECOG – Eastern Cooperative Oncology Group performance status. ∗∗∗∗ *p* < 0.0001. BAP1, BRCA1-associated protein 1; CCL2, C-C motif chemokine ligand 2; ELISA, enzyme-linked immunosorbent assay.

To evaluate the prognostic relevance of CCL2, we compared its performance with *BAP1* status, which has previously been associated with survival in mesothelioma. BAP1 retention was associated with significantly shorter survival in the validation cohort (median 10.8 vs 22.2 months, *p* < 0.0001; Fig. 2*A*), although no such difference was observed in the smaller discovery cohort (median 11.9 vs 11.0 months, *p* = 0.367; data not shown). In multivariate analysis of the validation cohort, BAP1 retention (Hazard Ratio [HR] = 1.84, *p* = 0.017), non-epithelioid histology (HR = 2.85, *p* < 0.001), and poor performance status at diagnosis (HR = 3.26, *p* < 0.001) were associated with shorter survival, whereas systemic therapy was associated with improved survival (HR = 0.58, *p* = 0.037) (Fig. 2*B*). CCL2 concentration in pleural effusion was not significantly associated with patient survival in either cohort after correction for clinical characteristics.

### CRISPR-Mediated BAP1 Deletion Reduces CCL2 Secretion In Vitro

**Figure 3:**
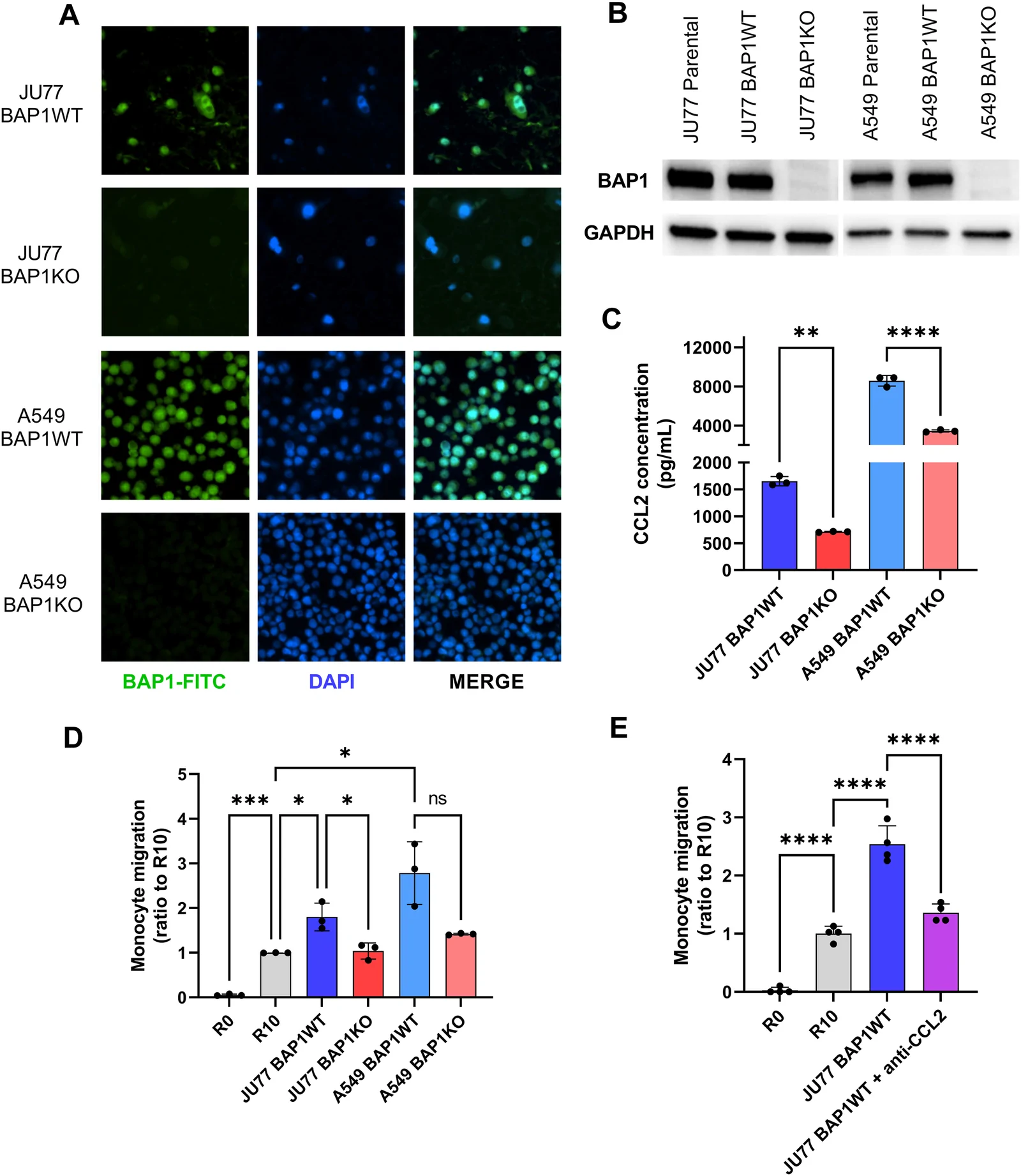
CRISPR-mediated BAP1 deletion reduces CCL2 secretion of in vitro mesothelioma and lung cancer cultures, and consequent CCL2-dependant monocyte recruitment. (A) Immunofluorescence imaging of CRISPR-edited JU77 (mesothelioma) and A549 (lung carcinoma) with BAP1-FITC (green) and DAPI (blue) confirming loss of BAP1 protein expression in BAP1WT and BAP1KO clones. (B) Western blotting of BAP1 in JU77 and A549 BAP1WT and BAP1KO, GAPDH used as a loading control. (C) CCL2 concentration in culture supernatant of BAP1WT and BAP1KO clones of JU77 and A549 cell lines, measured by ELISA after 72 hours in culture, compared using t-test. Data represents independent triplicate samples from each individual clone. Error bars represent mean and standard deviation. (D) Monocyte migration in response to supernatant from JU77 and A549 BAP1WT and BAP1KO cell lines, expressed relative to R10 control (RPMI containing 10% FCS). Supernatants were collected 144 hours post-seeding. Compared using one-way ANOVA, data represents three independent experiments using monocytes from independent healthy donors. (E) Monocyte migration in response to JU77 BAP1WT supernatant, with or without pretreatment with 4.5ug/mL anti-CCL2 neutralising antibody. Migration expressed as a ratio of R10 control. Compared using one-way ANOVA. Ns = *p* > 0.05, ∗ *p* < 0.05, ∗∗ *p* < 0.01, ∗∗∗ *p* < 0.001, ∗∗∗∗ *p* < 0.0001. ANOVA, analysis of variance; BAP1, BRCA1-associated protein 1; CCL2, C-C motif chemokine ligand 2; CRISPR, Clustered Regularly Interspaced Short Palindromic Repeats; DAPI, 4′,6-diamidino-2-phenylindole; ELISA, enzyme-linked immunosorbent assay; FCS, foetal calf serum; FITC, fluorescein isothiocyanate; GAPDH, Glyceraldehyde 3-phosphate dehydrogenase; KO, knockout; RPMI, Roswell Park Memorial Institute 1640; WT, wildtype.

To directly investigate whether BAP1 loss affects tumour-derived CCL2 secretion, we generated BAP1KO using CRISPR-Cas9 in JU77 mesothelioma and A549 lung carcinoma cell lines. Successful editing was confirmed by Sanger sequencing, which revealed homozygous frameshift mutations in both JU77 and A549 cell lines, and by complete loss of BAP1 protein as shown by immunofluorescence and western blotting (Fig. 3*A* and *B*). Clones that underwent the CRISPR process but retained BAP1WT were used as comparators.

CCL2 concentrations in cell culture supernatants collected after 72 hours were significantly lower in BAP1KO than in BAP1WT clones, in both JU77 (711 versus 1652 pg/mL, *p* < 0.0001) and A549 (3447 versus 8572 pg/mL, *p* < 0.0001) (Fig. 3*C*), indicating that BAP1 loss suppresses tumour CCL2 secretion in vitro.

### BAP1 Deletion Abolishes CCL2-Dependent Monocyte Recruitment

To evaluate the functional consequence of reduced CCL2 secretion, we assessed the capacity of BAP1KO tumour cell supernatants to recruit classical monocytes. Monocytes were isolated from independent healthy donor peripheral blood mononuclear cells and assessed in transwell migration assays. In JU77 cells, BAP1WT supernatants induced a mean migration value of 1.8 ± 0.3 relative to R10 control, whereas BAP1KO supernatants showed significantly reduced recruitment (1.03 ± 0.2; *p* = 0.03) (Fig. 3D). Similarly, in A549 cells, BAP1WT supernatants promoted higher monocyte migration (mean 2.8 ± 0.7) compared with BAP1KO supernatants (1.4 ± 0.02), although this difference did not reach statistical significance. (*p* = 0.075).

To determine whether CCL2 was the primary mediator of monocyte chemoattraction, BAP1WT JU77 supernatant was pre-incubated with anti-CCL2 neutralising antibody. This significantly reduced monocyte migration compared with untreated supernatant (2.54 ± 0.3 versus 1.36 ± 0.2, *p* < 0.0001) (Fig. 3*E*).

### *BAP1* Mutational Status is Associated With Increased Estimated Macrophage Abundance in TCGA Cohort

**Figure 4:**
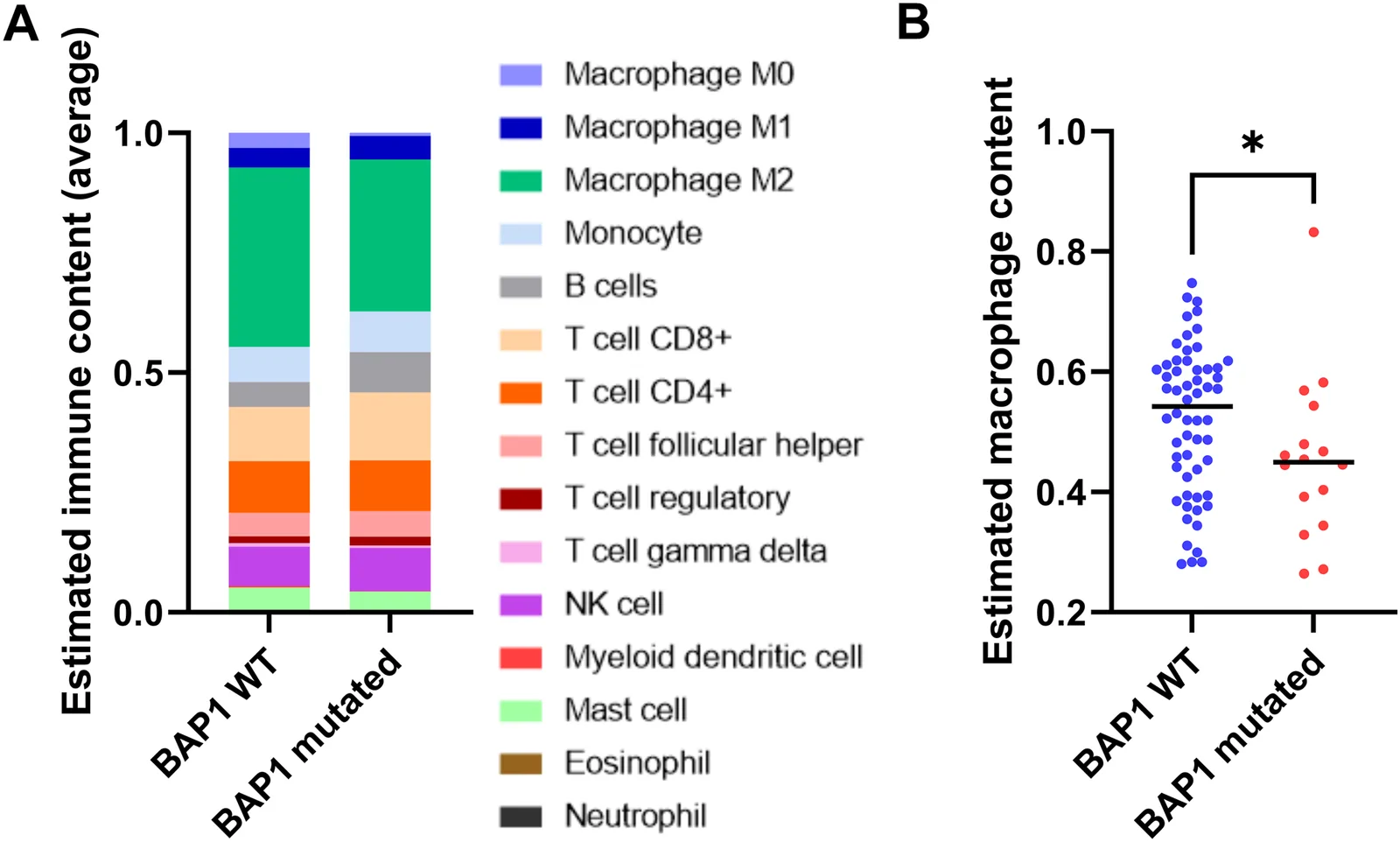
Immune cell content and CCL2 expression in the TCGA-MESO cohort. (*A*) Stacked bar chart of tumour immune cell content estimated by CIBERSORT in TCGA-MESO cohort (N = 74) patients. (*B*) Total macrophage content (sum of M0/M1/M2), estimated by CIBERSORT, in TCGA-MESO cohort patients grouped by *BAP1* mutational status. ∗ p < 0.05. BAP1, BRCA1-associated protein 1.

To further explore the relationship between BAP1 status, immune cell infiltration, and CCL2 regulation, we analysed publicly available data from TCGA MESO cohort (n = 74). Immune cell composition was estimated using CIBERSORT, which revealed that, on average, monocytes and macrophages comprised the majority of the immune infiltrate in *BAP1WT* tumours. Total macrophage content, calculated as the sum of M0, M1, and M2 subsets, was significantly higher in wild-type *BAP1* tumours than in mutant *BAP1* tumours (0.46 ± 0.12 versus 0.36 ± 0.10, *p* = 0.034) (Fig. 4*A* and *B*). No other immune cell subsets were significantly associated with BAP1 status. We did not observe significantly altered CCL2 mRNA expression in *BAP1*-mutated tumours (Supplementary Fig. 2).

## Discussion

Our study provides evidence that tumour BAP1 status is associated with differences in the immune composition of the pleural tumour environment in mesothelioma. BAP1 loss was associated with reduced levels of the chemokine CCL2 in pleural effusion samples across 2 independent cohorts. Soluble CCL2 concentrations did not themselves correlate with survival. Deletion of BAP1 reduced the secretion of soluble CCL2 from mesothelioma and lung cancer cells and abrogated the ability of tumour cultures to recruit monocytes in vitro. Complementary analysis of the TCGA-MESO cohort reported decreased estimated macrophage content in *BAP1*-mutant tumours. These findings suggest that BAP1 promotes tumour CCL2 secretion into the pleural space, which may then modulate the tumour environment through the recruitment of macrophages to the tumour.

CCL2 (also known as monocyte chemoattractant protein-1) is a key chemokine that mediates the recruitment of CCR2^+^ monocytes to sites of inflammation and tumour progression. Although few studies have directly measured soluble cytokines in the context of BAP1 loss, our findings are supported by a germline heterozygous BAP1 loss murine model, in which CCL2 levels were significantly reduced both at baseline and after asbestos exposure.^15^ In contrast, BAP1 loss in a renal cell carcinoma mouse model was associated with elevated tumour-derived CCL2 and other CCR5 ligands,^16^ suggesting a tissue-specific role for BAP1 in cytokine regulation.

In mesothelioma, few studies have directly assessed the impact of BAP1 loss on monocyte infiltration. Reduced CD14^+^ monocyte populations have been observed in BAP1-deficient tumours,^17^ and murine models similarly suggest an altered myeloid composition.^15^ Our data support a role for BAP1 in regulating monocyte recruitment: BAP1-deficient mesothelioma cells secrete less CCL2 and exhibit reduced monocyte chemoattraction in vitro, and *BAP1*-mutant tumours in the TCGA cohort show lower total macrophage abundance. These findings are consistent with transcriptomic studies showing that BAP1 loss is enriched in immune ‘cold’ mesothelioma subtypes, characterised by reduced cytotoxic T cell and interferon signalling and increased expression of immune checkpoint molecules such as LAG3 and VISTA,^18^^,^^19^ supporting the view that *BAP1* defines an immunologically distinct tumour phenotype.

Among the 22 soluble factors selected for analysis, only CCL2 was significantly associated with BAP1 status in pleural effusions. The panel was designed based on prior studies reporting associations between these cytokines and either BAP1 status or mesothelioma prognosis, primarily through gene expression analysis of tumour tissue. Notably, HMGB1 levels did not differ significantly between BAP1 subgroups, in contrast with previous work which has shown that BAP1 regulates the acetylation and secretion of HMGB1.^9^ No other cytokines in the panel were significantly associated with BAP1 status. The heterogeneous profile of soluble factors, including CCL2, may reflect other tumour genomic alterations, such as loss of Cyclin-Dependent Kinase Inhibitor 2A or Neurofibromatosis 2, playing a major role in regulating the secretome.

CCL2 has been investigated in mesothelioma for over a decade, with several studies highlighting its potential clinical relevance. It has been shown to be elevated in malignant pleural effusions and to distinguish mesothelioma from lung cancer-associated effusions.^20^ Experimental models further support a functional role for CCL2 in effusion formation.^21^ Furthermore, serum CCL2 is associated with disease stage; however, reports on the prognostic relevance of soluble CCL2 are mixed.^22^ In this cohort, CCL2 was not independently associated with survival after adjustment for clinical characteristics, whereas BAP1 status, histology, and performance status remained the dominant prognostic factors.

Limitations of this study include the lack of paired tumour tissue samples and effusion samples, which prevents the assessment of the relationship between effusion CCL2 and tumour monocyte or macrophage infiltration. The cohort is enriched for patients with epithelioid histology because epithelioid mesothelioma more often presents with pleural effusion than biphasic or sarcomatoid disease, where effusions can occur but tumour cells are less commonly shed.^23^ BAP1 loss is more frequent in epithelioid patients, resulting in a higher proportion of epithelioid histology than in BAP1-retained patients. An additional consideration is that BAP1 retention as determined by IHC does not necessarily reflect functional protein because various BAP1 missense variants can alter function without loss of nuclear localisation.^24^

Blockade of CCL2 has shown promise as an adjuvant to immune checkpoint blockade.^25^, ^26^, ^27^ As BAP1 has garnered growing interest as a biomarker for treatment stratification in mesothelioma, these findings raise the possibility that targeting the CCL2-CCR2 axis may be most effective in patients with BAP1 retention. In the context of mesothelioma, where current treatment options remain limited and immunosuppressive myeloid infiltration is a key barrier, targeting this pathway may hold particular promise in BAP1-defined subgroups in which CCL2 is selectively elevated.

## Conclusion

In conclusion, our findings suggest that BAP1 loss alters the immune landscape of pleural effusion in mesothelioma by downregulating CCL2. This may reduce the infiltration of monocyte-derived immunosuppressive macrophages, potentially leading to a more immunologically active tumour environment. These insights support the rationale for targeting the CCL2-CCR2 axis in BAP1-retained patients. Further studies should explore how BAP1 loss is associated with immune cell phenotype and functional state in pleural effusion, and whether CCL2 blockade may augment immunotherapy response in these patients.

## CRediT Authorship Contribution Statement

**Alistair Nash:** Conceptualisation, Data curation, Formal analysis, Investigation, Methodology, Project administration, Resources, Software, Validation, Visualisation, Writing - original draft, Writing - review & editing.

**Ebony Rouse:** Investigation, Methodology, Resources, Validation, Writing - review & editing.

**Tina Firth:** Resources, Writing - review & editing.

**Ian M Dick:** Conceptualisation, Formal analysis, Software, Writing - review & editing.

**Vanessa S Fear:** Writing - review & editing.

**Catherine A Forbes:** Writing - review & editing.

**Amber Louw:** Investigation, Writing - review & editing.

**Y C Gary Lee:** Conceptualisation, Writing - review & editing.

**Alec J Redwood:** Supervision, Writing - review & editing.

**Bruce W Robinson:** Supervision, Writing - review & editing.

**Jenette Creaney:** Conceptualisation, Data curation, Formal analysis, Funding acquisition, Methodology, Project administration, Supervision, Writing - original draft, Writing - review & editing.

## Disclosure

YCGL is an unpaid panel member for the Australian Mesothelioma Guideline Group. All authors have no further disclosures to report.
