# Supplementary material for: BAP1 Loss in Pleural Mesothelioma Is Associated With Reduced Soluble CCL2 in Patient Effusion, Abrogated CCL2-Mediated Monocyte Recruitment In Vitro
Source: JTO Clin Res Rep. 2026 Jul 10;7(10):101049. doi: 10.1016/j.jtocrr.2026.101049 (PMC13571929; doi:10.1016/j.jtocrr.2026.101049)
Supplement: Supplementary file 1 [file mmc1.docx]

**Supplementary Figure 1: Serum CCL2 concentrations in discovery cohort patients**

**(A)** Scatter plot of CCL2 concentrations in pleural effusions and matched serums from patients in the discovery cohort (N = 22). **(B)** Comparison of serum CCL2 concentrations between BAP1 retained (N = 11) and BAP1 loss (N = 11) patients in the discovery cohort.

**Supplementary Figure 2: CCL2 expression by BAP1 status in the TCGA-MESO cohort**

Normalised CCL2 expression in TCGA-MESO cohort tumours with wildtype BAP1 (N = 58) or BAP1 mutation (N = 16).
